# 

**Published:** 1917-12-01

**Authors:** 


Rates of Subscription: Twelve months, 6/6; Six months, 4/-; three months, 2/-, post free to any address in the United Kingdom. Abroad, Twelve months, 8/8; Six months, 5/-; Three months, 2/6, post free. THE HOSPITAL "Index" to Volume LXII., April 1917 to September 1917, is now ready, price 2½d. per copy, post free. Address The Manager, The Hospital, "The Hospital" Building, 28/29 Southampton Street, Strand, London, W.C. 2.

### Poor Law Infirmary Matrons' Association.

We regret that owing to pressure on our space the report of the annual meeting held on November 27 is unavoidably held over.

### Matrons' and Sisters' Appointments.

Dumfries and Galloway Royal Infirmary.—Miss Mary D. Crichton has been appointed lady superintendent. She was trained at the Liverpool Royal Infirmary, and has successively been ward sister, night sister, and assistant matron there.

Auxiliary Military Hospital, Helsby, Cheshire.—Miss M. Carswell has been appointed sister. She was trained at Guy's Hospital.

Crumpsall Infirmary, Manchester.—Miss Nellie Tranter and Miss Harriet Augusta Head have been appointed sisters. They were both trained at the above infirmary.

Fusehill War Hospital, Carlisle.—Miss J. McCreath, Miss Emily Miller, Miss M. E. Wheaton, Miss K. A. Smith, Miss Lydia Taylor, Miss E. de V. Fletcher, Miss J. Cochran, Miss Catherine Dean, Miss J. F. H. Cherry, Miss Esther Poland, Miss Catherine M. Mercer, and Miss Minnie Cook have been appointed sisters. Miss Wheaton and Miss Poland were trained at St. Bartholomew's Hospital, London. The former has held posts at Lady Roberts' Hospital, India, Fulham War Hospital, and the Auxiliary Military Hospital, Kington, Hereford. Miss Poland has been sister at Thorncombe Military Hospital, Bramley, Surrey, and at Bankfield Military Hospital, Workington. Miss Taylor and Miss Dean were trained at the Cumberland Infirmary, Carlisle. Miss Taylor has been sister at Beckett Hospital, Barnsley, Stockport Infirmary, and Plaistow Fever Hospital; matron of Leith Public Health Hospital and Grampian Sanatorium, Kingussie; and sister T.F.N.S. 3rd Northern General Hospital. Miss Dean has been sister at the South London Hospital for Women. Miss J. McCreath was trained at Leith General Hospital and the City Hospital, Edinburgh, and has been nursing in the Q.A.I.M.N.S.R. in military hospitals at Home and in France. Miss Miller was trained at Glasgow Royal Infirmary, and has been sister at the Poor-Law Hospital, Smithston, Old Markland Hospital, Coatbridge, and the Bangour Military Hospital, Edinburgh. Miss Smith was trained at Arbroath Infirmary, and has been sister and matron of the Oxygen Hospital, London, and has nursed at Queen Mary's Naval Hospital, Southend. Miss Cherry was trained at the Royal Victoria Hospital, Belfast. Miss Fletcher was trained at Guy's Hospital, and has been sister at Queen Mary's Hospital for Children, Carshalton, and at Graylingwell War Hospital, Chichester. Miss Cochran was trained at Merryflatts Hospital, Govan, and has been sister at Glasgow Eye Hospital, Birmingham Eye Hospital, and Townley's Military Hospital, Farnworth. Miss Mercer was trained at the Western Infirmary, Glasgow, and has been sister at the Dundee War Hospital and at the Lochee Red Cross Hospital, Dundee. Miss Cook was trained at Stobhill Hospital, Glasgow, and has held posts at Wharncliffe Military Hospital, at Salonika, and at Queen Mary's Military Hospital, Whalley.

General Hospital, Leith.—Miss J. S. McIntyre and Miss Gertrude Conolly have been appointed sister and night sister respectively. The former was trained at the Glasgow Western Infirmary, and has been ward sister at Manchester Children's Hospital, staff nurse at Ransome Sanatorium, Mansfield, and sister at the Military Hospital, Sutton Veny, Wilts. Miss Conolly was trained at Oldham Royal Infirmary and Carr Gate Hospital, Wakefield, and has been sister at Edinburgh Hospital for Women, at the Royal Hospital for Sick Children, Edinburgh, and at the Red Cross Hospital, Troon. She has also done war work in Egypt.

Home Sanatorium, West Southbourne, near Bournemouth.—Miss Julia Swallow has been appointed sister. She was trained at the Northampton General Hospital, has been sister of the training-school and night sister at St. Luke's Hospital, Bradford; matron-nurse of the Cottage Hospital, Crewkerne; and temporary home sister at Salisbury General Infirmary.

Jaffray Hospital, Erdington, Birmingham.—Miss Ada Turner has been appointed sister. She was trained at the Leicester Royal Infirmary, and has held posts at the British Cottage Hospital, Algiers, and the Morant War Hospital, Brockenhurst, Hants. She has also done private nursing.

Joyce Green Hospital, Dartford, Kent.—Miss Amy Holmes has been appointed sister. She was trained at the Adelaide Hospital, Dublin, and has been superintendent sister at Queen Mary's Hospital, Carshalton. She has also done private nursing.

North Evington Military Hospital, Leicester.—Miss Ethel M. Lloyd, T.F.N.S., has been appointed sister at this hospital, where she has been serving for over two years. She was trained at the Royal Infirmary, Bristol, and the Children's Hospital, Birmingham. She was in France at the beginning of the war.

Royal United Hospital, Bath.—Miss E. Wilks has been appointed sister. She was trained at Guy's Hospital, and has been sister at the Royal Surrey County Hospital, Guildford, and at the Swansea General and Eye Hospital.

**NOTE.—Particulars of Appointments for insertion in this column will be welcomed.**

### Passing Events and Topics.

### "Winter's Pie."

"Winter's Pie, 1917," has made its appearance, the price remaining the same as usual—one shilling. It includes a substantial collection of contributions by eminent authors and artists.

### A Yuletide Fair.

St. Nicholas' Home for Raid-Shock Cases Among School Children is quite the most recent charity; in its aid a Yuletide Fair will be held at 26 Great Cumberland Place, W., the house of Sir Francis and Lady Lloyd, on Wednesday, December 5, at 3 p.m.

### Royal College of Surgeons.

A first series of a course of lectures on the Anatomical and Physiological Principles underlying the Treatment of Injuries to Muscles, Joints, and Bones will be delivered by Professor Arthur Keith, Conservator of the Museum of the Royal College of Surgeons of England, in the Theatre of the College, Lincoln's Inn Fields, on Mondays, Wednesdays, and Fridays, from November 23 to December 14. The second series will be given in January.

### An Infant Welfare Campaign.

A Provisional Committee has been formed in London, of which Lord Plunkett is chairman, and has invited Dr. Truby King, of New Zealand, to come to England, and take an active part in the Infant Welfare campaign now being organised. Dr. King is well known as the founder of the Royal New Zealand Society for the Health of Women and Children. It is expected that he will reach England about next February.

### The Royal Hospital for Incurables.

Founders' Day will be celebrated at the Royal Hospital for Incurables on Tuesday, December 4, when the Board of Management and the patients will be "At Home" from 2 to 6 p.m.

### A New Auxiliary Hospital at Llandaff.

St. Michael's Theological College, Llandaff, has been opened as an auxiliary military hospital for wounded and invalided officers. It is the biggest hospital of its kind in Wales. Provision is made for the accommodation of seventy-five patients, and in the event of emergency the number of beds can be increased to a hundred. Miss E. M. Russle is matron of the institution, and Dr. Naish, of Cardiff, is in charge as medical officer.

